# New Insights in Respiratory Diseases

**DOI:** 10.3390/biomedicines14020427

**Published:** 2026-02-13

**Authors:** Diego Bagnasco, Benedetta Bondi

**Affiliations:** 1 IRCCS Azienda Ospedaliera Metropolitana, 16132 Genoa, Italy; benedetta.bondi@edu.unige.it; 2 Department of Internal Medicine, University of Genoa, DiMI, 16126 Genoa, Italy

In recent years, pulmonology has undergone a profound transformation, perhaps the most significant since the birth of modern respiratory medicine. It is a change that does not stem from a single discovery but from an awareness of the need to bring together different disciplines, such as immunology, mechanobiology, metabolomics, genetics, digital epidemiology, and behavioural medicine, to manage the patient. The articles collected in this Special Issue show how much this change is already underway and how it is redefining the way we understand, diagnose, and treat respiratory diseases.

The work of Panek and colleagues on asthma management in Europe is emblematic [1]. Although the use of inhaled drugs is crucial in the treatment of patients with asthma, and the use of the ICS/LABA/LAMA triple therapy has been supported by solid clinical evidence for several years now and is recommended by the GINA guidelines for uncontrolled patients, it continues to be underused. This study confirms, once again, a phenomenon that comes as no surprise to those who have been studying therapeutic inertia in asthma for years, that of the low use of LAMAs in asthma, and the reduced flexibility and variability of the therapy itself, contravening one of the principles that the guidelines themselves suggest, that of step-up and step-down therapy. This habit is not only a limitation to the correct treatment of patients, but it is also a major source of increased healthcare expenditure [2,3]. This, in the era of monoclonal therapies for asthma, of clinical remission, must be taken into account in order not to replace biological drugs; quite the opposite, to consider triple therapy an important adjuvant to monoclonal antibodies so that the goal of clinical remission is achieved more easily and more durably [3,4,5]. In addition, the opportunity of modulating therapy and providing a wide range of drugs makes it possible to evaluate the possibility of remodulating inhalation therapy, either by managing the dosage of ICSs, reducing it but maintaining two other drugs (LAMA and LABA), or by discontinuing LAMA, with a view to a step-down, when possible [6]. Alongside this, leaving asthma and progressing towards COPD and pulmonary fibrosis, Cantor’s article introduces a fascinating conceptual paradigm: percolation theory applied to the lung [7]. The central idea is that lung tissue behaves like a complex network, in which the connectivity between collagen and elastin fibres determines the stability of the entire structure. When chronic inflammation alters this network, the system can exceed a critical threshold and embark on one of two pathological trajectories: emphysema or fibrosis. Increased tissue stiffness, driven by enzymes such as LOX and LOXL2, has been identified as a key driver of fibrotic progression [8,9]. The literature not only points to a role of these two enzymes in emphysema and the development of pulmonary fibrosis but also in the worse prognosis of squamous cell carcinoma of the lung [10]. In summary, the fate of the inflamed lung depends on how crosslinking alters network connectivity: loss of links leads to the development of emphysema; excess of links to fibrosis. Between these extremes are the mixed phenotypes, explained as states close to the critical threshold.

The contribution by He and colleagues on the metabolic reprogramming of fibroblasts fits perfectly into this picture [11]. Evidence that SIK2 amplifies glycolysis and supports fibroblast activation confirms a growing line of research identifying cell metabolism as a crucial therapeutic node in pulmonary fibrosis. Recent studies converge to show that specific alterations in metabolism, such as glycolysis, mitochondrial metabolism, amino acid metabolism, and lipid metabolism, are not simply consequences of the disease but active drivers of fibrotic progression. Damaged alveolar epithelial cells enter a state of metabolic stress characterised by mitochondrial dysfunction, reduced oxidative capacity, and increased glycolysis. This metabolic shift alters their repair capacity and promotes profibrotic signals that activate fibroblasts. In parallel, the fibroblasts themselves undergo a real metabolic reprogramming: they increase glycolysis to support the production of extracellular matrix, depend more on glutamine and arginine, and show a profoundly altered lipid metabolism [12]. A recent study has also shown that the metabolism of amino acids, in particular glutamine, proline, and arginine, is essential for collagen synthesis and fibroblast activation, making it a direct therapeutic target [13].

Moving away from chronic and developmental diseases to the respiratory infections front, the work of Mudgal et al. on the impact of RSV in adults with pulmonary hypertension highlights an often underestimated aspect: vascular and cardiopulmonary fragility as a determinant of the severity of viral infections [14]. Several studies have shown that RSV infection in adults is associated with cardiovascular complications, re-hospitalisations, and increased mortality, indicating significant endothelial involvement [15]. Direct comparisons between RSV and influenza show that both viruses can induce vascular instability, with an increase in thrombotic and cardiac events during hospitalisation [16]. Finally, large-scale longitudinal investigations confirm that patients hospitalised for RSV have an elevated risk of complications both during their hospital stay and in the following months, consistent with persistent endothelial dysfunction [17]. Overall, the evidence suggests that the endothelium is a central mediator of clinical severity: when viral infection damages it, the risk of cardiovascular complications, the need for hospitalisation, and adverse outcomes increase dramatically, spurring the scientific community and those involved in cardiorespiratory disease to increasingly evaluate preventive options, such as RSV vaccines, in their patients.

Continuing through the respiratory diseases of early life, the work of Dorgham et al. on the LPCAT1 rs9728 variant reminds us of how genetics contributes to respiratory vulnerability in childhood [18]. Still concerning the early stages of life, Ying and colleagues focused on the use of glucocorticoids in paediatric pneumonia, stressing another crucial element: the need to personalise therapies [19]. The use of causal inference models to identify patients who actually benefit from corticosteroids is an important step towards more precise respiratory medicine. The study shows that glucocorticoids do not have a uniform effect in infants and young children with severe pneumonia. To identify these subgroups, the authors used an advanced machine learning approach, the causal forest, to estimate the individual treatment effect for each patient. The use of AI methods made it possible to understand which variables drive the best or worst response to the steroid, highlighting how machine learning can make it possible to move from a ‘one-size-fits-all’ use of glucocorticoids to a personalised approach, identifying who can really benefit and reducing unnecessary or potentially harmful treatments.

Finally, the study by Ianosi et al. on young adults highlights a worrying phenomenon: early damage of the small airways in smokers, including e-cigarette users, a finding that is perfectly consistent with the recent literature [20]. The available evidence converges on one very clear point: e-cigarettes are not harmless to the lungs. Several studies show that e-cig vapour directly damages the airway epithelium, altering the barrier and ciliary function, and increasing oxidative stress. Other work shows that vapour components interfere with surfactant structure and function, impairing alveolar stability. In parallel, exposure to vapour, even without nicotine, induces a marked inflammatory response, with increases in IL-6, mucins, and pro-inflammatory mediators. It is therefore important to remember that not only ‘traditional’ cigarettes but also e-cigs have been associated with epithelial dysfunction, surfactant alterations, and inflammation of the peripheral airways [21,22,23,24].

Looking at these contributions together, a clear message emerges: pulmonology is entering an era in which the distinction between prevention, diagnosis, and treatment is becoming increasingly blurred. Machine learning technologies make it possible to predict response to treatment; mechanobiology and metabolomics offer new therapeutic targets; genetics identifies early vulnerabilities; advanced imaging reveals previously invisible structural patterns; and understanding risk behaviour in young people enables more effective preventive interventions (Figure 1).

## Figures and Tables

**Figure 1:**
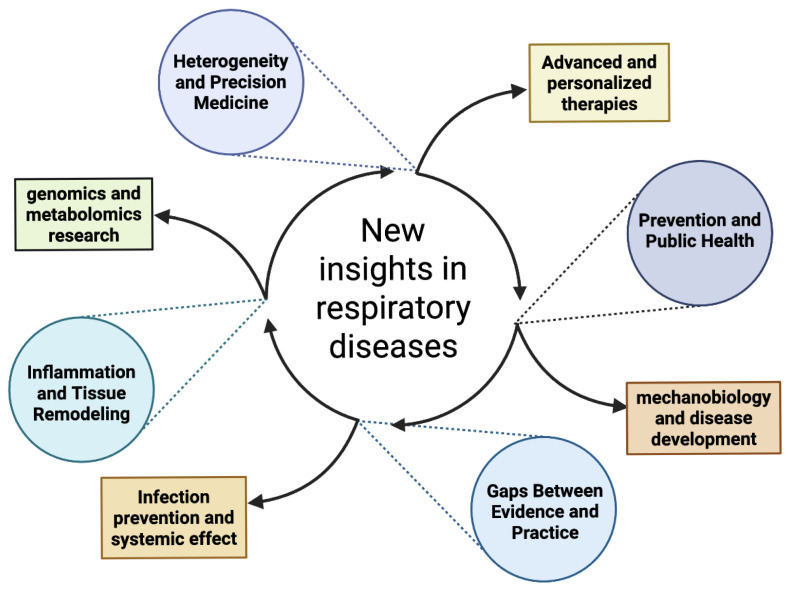
Principal strategies and issues for new insights into respiratory diseases.

## References

[1] Panek M., Breyer-Kohansal R., Steiropoulos P., Kopač P., Knopczyk M., Dębowski T., Janson C., Kupczyk M. (2025). Challenges Pertaining to the Optimization of Therapy and the Management of Asthma—Results from the 2023 EU-LAMA Survey. Biomedicines.

[2] Bloom C.I., de Preux L., Sheikh A., Quint J.K. (2020). Health and cost impact of stepping down asthma medication for UK patients, 2001–2017: A population-based observational study. PLoS Med..

[3] Bagnasco D., Ansotegui I., Baiardini I., Benfante A., Bernstein J., Bikov A., Bondi B., Boulet L., Panaitescu C., Canonica G. (2024). Triple inhaled therapy in asthma: Beliefs, behaviours and doubts. Pulm. Pharmacol. Ther..

[4] Braido F., Tiotiu A., Guidos-Fogelbach G., Baiardini I., Cosini F., de Sousa J.C., Bikov A., Novakova S., Labor M., Kaidashev I. (2022). Manifesto on inhaled triple therapy in asthma: An Interasma (Global Asthma Association–GAA) document. J. Asthma.

[5] Canonica G.W., Bagnasco D., Bondi B., Varricchi G., Paoletti G., Blasi F., Paggiaro P., Braido F. (2024). on behalf of the SANI study group. SANI Clinical Remission definition: A useful tool in Severe Asthma management. J. Asthma.

[6] Jackson D.J., Heaney L.G., Humbert M., Kent B.D., Shavit A., Hiljemark L., Olinger L., Cohen D., Menzies-Gow A., Korn S. (2024). Reduction of daily maintenance inhaled corticosteroids in patients with severe eosinophilic asthma treated with benralizumab (SHAMAL): A randomised, multicentre, open-label, phase 4 study. Lancet.

[7] Cantor J. (2026). Percolation Forces in Lung Inflammation: Determining the Path 2 to Emphysema or Fibrosis. Biomedicines.

[8] Ma H.-Y., Li Q., Wong W.R., N’diaye E.-N., Caplazi P., Bender H., Huang Z., Arlantico A., Jeet S., Wong A. (2023). LOXL4, but not LOXL2, is the critical determinant of pathological collagen cross-linking and fibrosis in the lung. Sci. Adv..

[9] Öztay F., Besiktepe N., Ersen E., Öztay F., Besiktepe N., Ersen E. (2017). Role of lysyl oxidases in pathogenesis of pulmonary emphysema. Eur. Respir. J..

[10] Cao L., Zhong J., Liu Z., Jiang J., Zhu C., Liu F., Wang B. (2024). Increased LOXL2 expression is related to poor prognosis in lung squamous cell carcinoma. J. Thorac. Dis..

[11] He J., Dong R., Yue H., Zhang F., Dou X., Li X., Li H., Zhang H. (2025). SIK2 Drives Pulmonary Fibrosis by Enhancing Fibroblast Glycolysis and Activation. Biomedicines.

[12] Dai T., Liang Y., Li X., Zhao J., Li G., Li Q., Xu L., Zhao J. (2025). Targeting alveolar epithelial cell metabolism in pulmonary fibrosis: Pioneering an emerging therapeutic strategy. Front. Cell Dev. Biol..

[13] Zheng H., Zhang L., Wang C., Wang Y., Zeng C. (2025). Metabolic dysregulation in pulmonary fibrosis: Insights into amino acid contributions and therapeutic potential. Cell Death Discov..

[14] Mudgal M., Bhatnagar A.R., Vasudevan A.K., Gajendiran A.P., Gondhi V., Balaji S., Murugan S.K., Gunasekaran K. (2025). The Impact of Pulmonary Hypertension on Hospitalization Risk in Adults with Respiratory Syncytial Virus Infection. Biomedicines.

[15] Yayan J. (2025). Impact of RSV infection on mortality, rehospitalization, and long-term pulmonary, cardiovascular, and functional outcomes in hospitalized adults: A systematic review and meta-analysis. Virol. J..

[16] Inoue N., Nagai H., Fushimi K. (2025). Severity and outcomes of adult respiratory syncytial virus inpatient compared with influenza: Observational study from Japan. Infect. Dis..

[17] Descamps A., Lenzi N., Galtier F., Lainé F., Lesieur Z., Vanhems P., Amour S., L’HOnneur A.-S., Fidouh N., Foulongne V. (2021). In-hospital and midterm post-discharge complications of adults hospitalised with respiratory syncytial virus infection in France, 2017–2019: An observational study. Eur. Respir. J..

[18] Dorgham S., Yahia S., Shahin D., Eita A.M., Toraih E.A., Elshazli R.M. (2025). Association of LPCAT1*rs9728 Variant with Reduced Susceptibility to Neonatal Respiratory Distress Syndrome. Biomedicines.

[19] Ying Z., Ge H., Han W., Hu G., Zhu Z., Wang J., Song L., Qu D., Jin Z. (2025). Assessing the Heterogeneous Treatment Effects of Glucocorticoids in Infants and Toddlers with Severe Pneumonia. Biomedicines.

[20] Ianosi E.-S., Ianosi R.-I., Finta H., Lefter R.-A., Văsieșiu A.M., Huțanu D., Ianosi M.-B. (2026). The Influence of Smoking on Respiratory Function in Medical Students at the University of Medicine, Pharmacy, Science and Technology of Târgu-Mureș. Biomedicines.

[21] Gellatly S., Pavelka N., Crue T., Schweitzer K.S., Day B.J., Min E., Numata M., Voelker D.R., Scruggs A., Petrache I. (2020). Nicotine-Free e-Cigarette Vapor Exposure Stimulates IL6 and Mucin Production in Human Primary Small Airway Epithelial Cells. J. Inflamm. Res..

[22] Garavaglia M.L., Bodega F., Porta C., Milzani A., Sironi C., Dalle-Donne I. (2023). Molecular Impact of Conventional and Electronic Cigarettes on Pulmonary Surfactant. Int. J. Mol. Sci..

[23] McAlinden K.D., Eapen M.S., Lu W., Sharma P., Sohal S.S. (2020). The rise of electronic nicotine delivery systems and the emergence of electronic-cigarette-driven disease. Am. J. Physiol. Lung Cell Mol. Physiol..

[24] Yang X., Che W., Zhang L., Zhang H., Chen X. (2025). Chronic airway inflammatory diseases and e-cigarette use: A review of health risks and mechanisms. Eur. J. Med. Res..

